# Interaction of potato-derived RG-I with galectin-3 and gene regulation in human mesenchymal stem cells

**DOI:** 10.1186/s12896-025-01030-z

**Published:** 2025-10-23

**Authors:** Amir Mukhtar, Athina Giannoudis, Bodil Jørgensen, Dongmei Wu, Xuan Liu, Kamal Babikeir Elnour Mustafa, Gudveig Cecilie Gjerde Gjengedal, Oluwatobi Adegbite, Anna Mieszkowska, Lu-Gang Yu, Katarzyna Gurzawska-Comis

**Affiliations:** 1 Institute of Life Course and Medical Sciences, School of Dentistry, University of Liverpool, Liverpool, UK; 2 Department of Plant and Environmental Sciences, University of Copenhagen, Copenhagen, Denmark; 3 Department of Microbial Pathogenesis, Yale University, New Haven, CT USA; 4 Shared Research Facilities, Centre for Genomic Research, University of Liverpool, Liverpool, UK; 5 Department of Clinical Dentistry, University of Bergen, Bergen, NO-5020 Norway; 6 Institute of Systems, Molecular and Integrative Biology, University of Liverpool, Liverpool, UK; 7 Department of Evolutionary Immunology, Institute of Zoology and Biomedical Research, Faculty of Biology, Jagiellonian University, Krakow, Poland; 8 Department of Dentistry and Oral Health, Section of Oral and Maxillofacial Surgery and Oral Pathology, Aarhus University, Vennelyst Boulevard 9, Aarhus, C DK-8000 Denmark; 9 University of Liverpool, Institute of Life Course and Medical Sciences, Department of Musculoskeletal and Ageing Science, Liverpool, United Kingdom

**Keywords:** Plant-derived nanoparticles, Rhamnogalacturonan-I (RG-I), Bone regeneration, Transcriptomic profiling, Galectin-3, In-silico analysis, Inflammatory response

## Abstract

**Background:**

Mesenchymal stem cells (MSC) play a vital role in bone regeneration. Poly-(l-lactide-co-ε-caprolactone) scaffolds functionalised with modified rhamnogalacturonan-I (RG-I) potato pectin, can modulate inflammation and promote bone regeneration in-vitro and in-vivo by modulating the action of the galactoside-binding galectin-3. In this study, we determined the binding affinity to galectin-3 of potato unmodified RG-I (PU) and its arabinose-deficient form (PA) and investigated the transcriptomic effects of PA treatment on human MSCs evaluated through in-silico pathway analysis in relation to galectin-3.

**Methods:**

The binding affinity of RG-I (PU and PA) to galectin-3 was assessed by tryptophan fluorescence spectroscopy. Human MSC (hMSC) isolated from the bone marrow of elderly patients (> 60yrs), were analysed by transcriptomic profiling. The effect of PA surface coating on gene expression was assessed in-silico by the ingenuity pathway analysis (IPA).

**Results:**

RG-I binds to galectin-3 with a binding affinity of 8.66 × 10^− 6^ M. Galactose sidechains of PA resulted in ~ 10-fold increased binding of RG-I to galectin-3 compared with PU. Forty-two genes were found to be differentially expressed (DE) in hMSCs cultured on PA surface coating, with a false-discovery rate (FDR) < 0.1. IPA of these DE genes including LGALS3, encoding for galectin-3, showed enrichment for organismal disorders-abnormalities, connective tissue and skeletal muscular disorders, immunological and inflammatory diseases, and identified three gene networks: infectious diseases, immune & inflammatory response and cell cycle control.

**Conclusions:**

This study indicates that modified potato pectin (PA) can regulate the expression of a broad range of genes, including galectin-3, that are involved in cellular, inflammatory, and immunological functions of hMSCs and may potentially be used to promote bone regeneration via modulation of inflammation.

**Supplementary Information:**

The online version contains supplementary material available at 10.1186/s12896-025-01030-z.

## Introduction

Bone possesses the intrinsic capacity for regeneration as part of the repair process in response to injury, as well as during skeletal development or continuous remodelling during adult life. Recent research has shown that nanoparticles can promote bone regeneration [1, 2]. Rapid advances are being made in tissue engineering using organic nanoparticles [3, 4], due to its mimicking structure to human biomolecules. Plant-derived nanoparticles, Rhamnogalacturonan-I (RG-I) is a polysaccharide with physical and chemical properties offering functionalisation of various biomaterials, such as titanium and synthetic scaffolds (co-polymer) [5–8]. A recent study developed immune-instructive copolymer scaffolds functionalised with RG-I isolated from potato, with two different structures, unmodified potato RG-I (PU) and modified with only galactan sidechains, called potato dearabinated RG-I (PA). Compared to PU, PA showed enhanced osteogenic metabolic activity and bone regeneration [9]. The functional part of PA that modulates immune responses has been shown to be the galactan sidechain, which binds to the galactoside-binding galectins [10, 11]. Both Galectins 1 (LGALS1) and 3 (LGALS3) mRNA expression and Galectin-3 protein expression was shown early to be downregulated in the neutrophils and macrophages cultured on the PA scaffold, suggesting a possible role of RG-I-galectin interaction in damping inflammatory response of immune cells [10, 12].

Mesenchymal stem cells (MSCs) play a vital role in bone regeneration. These are multipotent stem cells with the capability to support osteogenesis by differentiating into cells like osteoblasts. The osteogenic differentiation potential of MSCs is essential for their role in bone regeneration, as they can form new bone tissue and contribute to the repair of bone defects [13, 14]. They have also been reported to interact with numerous other proteins, including galectins, to support overall recovery during injury by regulating MSC osteogenic differentiation and bone regeneration [15–17]. An early study has shown that deletion of galectin-1 caused bone loss in mice due to impaired osteogenic differentiation potential of bone marrow-derived MSCs [17]. Galectin-3 has also been reported to orchestrate physiological and pathophysiological processes within bone regeneration in the human body and is expressed in various tissues, including bone, where it is considered a marker of chondrogenic and osteogenic cell lineages and a therapeutic target [18]. Galectin-3 has been implicated in age-dependent and diabetes-associated bone fragility and has a regulatory role in inflammatory bone and joint disorders, making it a possible therapeutic target [19]. It can commit MSCs to the osteoblastic lineage and favour trans-differentiation of vascular smooth muscle cells into an osteoblast-like phenotype, which opens a new area of interest in bone and vascular pathologies [20, 21].

In this study, we determined the galectin-3 binding affinity of RG-I and its arabinose-deficient form (PA) and conducted in-silico analysis of their effect on gene expression in human MSCs.

## Methods

### Determination of the binding affinity of PA and PU to Galectin-3

The binding affinity of PA and PU to galectin-3 was determined by tryptophan fluorescence spectroscopy (TFS) [22]. Human recombinant galectin-3 at a concentration of 10µM was equilibrated in phosphate-buffer saline (PBS) for 10 minutes under continuous stirring before titration with PA (0 to 1.2µM) or PU (0 to 3.4µM) solutions. Control buffer titrations were conducted in parallel to determine background fluorescence for each experiment. The experiments were conducted using a CARY Eclipse Fluorescence Spectrophotometer (Varian Inc., Santa Clara, California, USA) with a 3 ml quartz cuvette. The excitation wavelength was set at 285nm, and emission spectra were recorded from 300-500nm with a slit width of 5nm. Temperature was maintained at a constant 25°C using an external thermostatic water circulator. The binding affinity (Kd) and standard error were calculated using R (Version 4.0.1) with the ‘One Site-Specific Binding’ model (Y = Bmax * X / (KD + X) through non-linear curve fitting, where X is the ligand concentration, Y is the fluorescence intensity, Bmax is the maximum specific binding, and KD is the equilibrium binding constant, which typically provides a robust assessment of compound-ligand interactions [22].

### In vitro testing of gene regulation on hMSCs cultured on PA-coated surface

The hMSCs were harvested from the bone marrow of two females, over 60 years old, in triplicates. The study was carried out in accordance with the Declaration of Helsinki and following Ethical Approval by Regional Committee for Medical and Health Research Ethics in Norway (2013/1248/REK sør-øst C). Informed consent to participate was obtained from both participants. Cells were expanded and cultured for two weeks before in-vitro testing. hMSCs were cultured on PA-coated (test group) and uncoated tissue culture polystyrene surface plates (TCPS) (control group) for 7 days. The PA was selected for in-vitro testing based on results from previous studies [8, 9, 23]. Culture medium (MEM) (Gibco, Darmstadt, Germany) containing 18% foetal bovine serum (FBS) (Biochrom, Berlin, Germany), antibiotic (100 mg/L streptomycin and 100U/ml penicillin) (Biochrom), and 10 ml/L l-glutamine (Biochrom) was changed every 3 days. Total RNA was isolated using the RNA mini kit (Qiagen, Germany) and quality assessment was performed using the Agilent 2100 Bioanalyzer.

### Transcriptomic profiling and in-silico analysis

Strand-specific libraries were constructed using a TrueSeq RNA sample preparation kit (Illumina, San Diego, CA, USA), and RNA sequencing was carried out using an Illumina Novaseq 6000 instrument. The raw data were processed, and the counts were normalised using the DESeq2 software that applies the Wald test to calculate the p and the p-adjusted values using the Benjamini-Hochberg (BH) adjustment (https://bioconductor.org/packages/release/bioc/html/DESeq2.html.) [24]. The expression of the genes was calculated by the DESeq2 software and differentially expressed genes (DEGs) between PA and controls (non-coated TCPS) were determined using the MA-plot-based method in the DEGseq package using a q-value [false-discovery rate (FDR)] < 0.1 and log2 fold-change (FC) > 0.5 as cut-off.

The Ingenuity Pathway Analysis system (IPA system; Qiagen), which includes canonical pathway analysis, disease and function, regulator effects, upstream regulators, and molecular networks, was used for subsequent bioinformatics analysis. For each analysis, a p-value < 0.05 and FDR < 0.1 was set as the threshold.

### Coating stability of RG-I

RG-I was modified as previously described [8]. Elisa plates with 96 wells were coated with PA or PU at 1 mg/mL in 10mM bicarbonate. In a 96-well plate 50 µl of either PA or PU is added for each well with 6 replicates for each day sampling. In the control wells 10mM NaHCO3 solution is added. The 96-well plate is incubated overnight at 4 °C on a rotation plate at 100 rpm. For each day the 96-well plates are washed with milliQ water with new samples for 0, 1, 2, 3, 4, 5 and 6 days respectively and the solution is collected from each well and stored at 4 °C until further analysis. ELISA is performed on the coated surface after each time point for each well to investigate the amount of coating staying on the surface after washing. LM5 antibody (Plant Probes, Leeds, UK), recognizing galactan sidechains on RG-I, is used for the ELISA test. The wells are blocked for 15 min with 200 µl 5% skimmed milk in PBS, pH 7.2 and then LM5 diluted 1:10 is added to the wells and incubated on a shaker for two hours. The wells are washed three times. Subsequently 1:1000 diluted AP-conjugated secondary antibody, anti-rat IgG in 5% of skimmed milk is added and incubated on a shaker for two hours. The wells are washed three times with PBS and once with water. The phosphate substrate is added to each well and placed on a shaker for 10 min. The reaction can be detected at 450 nm with 570 nm as background on a spectrophotometer. The resulting absorbance corrected for the control is compared and standard deviation made for alle timepoints.

## Results

### RG-I modification, characterisation and coating stability

In comparison to PU, the molecular weight (Mw) of PA was reduced by 15% [PU Mw: 2017.2 (± 0.7%) kDa vs. PA Mw 1710.8 (± 0.2%) kDa] corresponding to the loss of the arabinan sidechains.

**Fig. 1:**
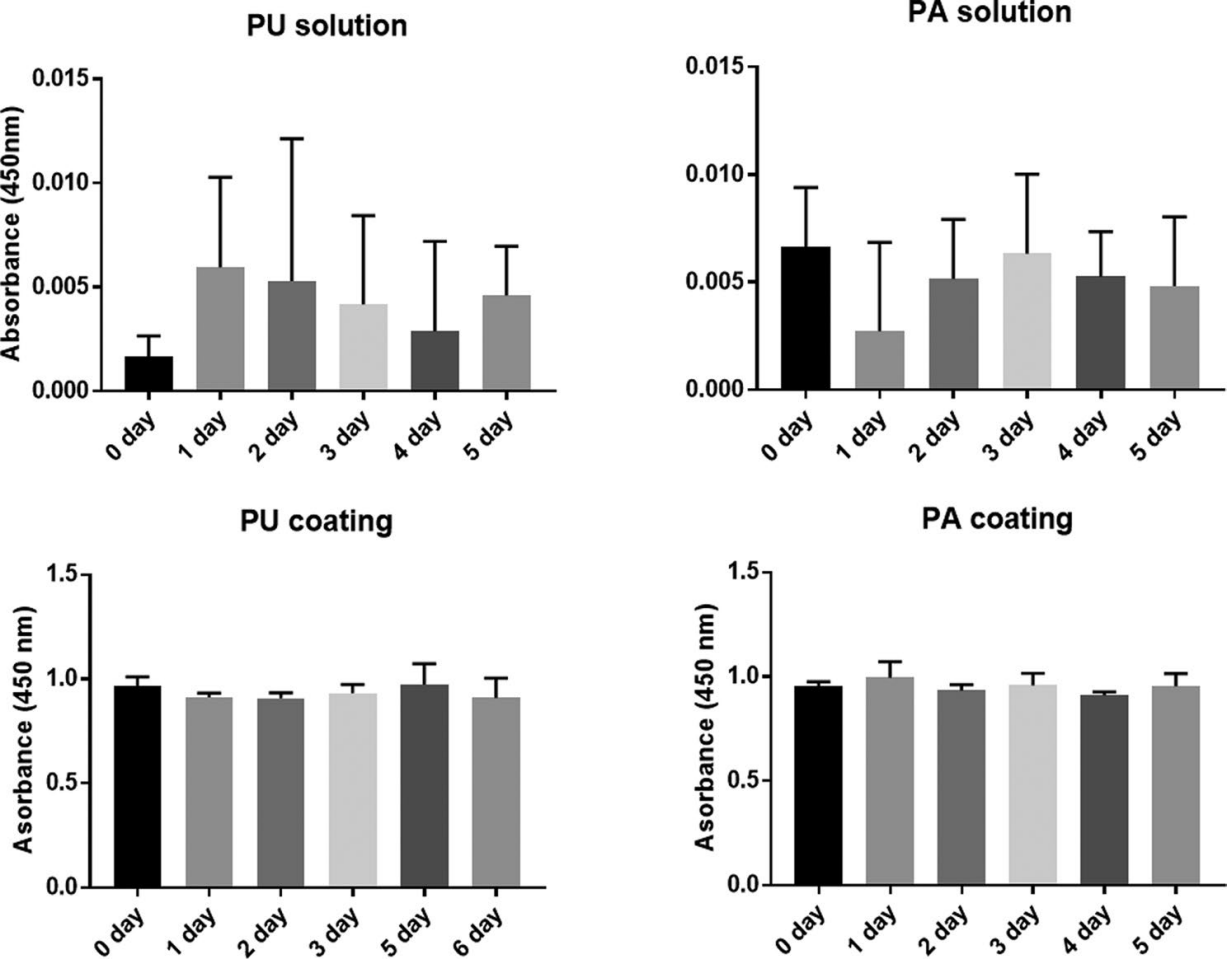
Analysis of RG-I stability by ELISA. PU and PA in solution (top panel) and on coated TCPS plates (bottom panel) showed no significant differences over a period of 6 days. However, lower absorbance of RG-I was observed in solution (top) than on coated TCPS plates (bottom) suggestive of better RG-I stability on TCPS plates

The stability of RG-I in solution and on coated TCPS plates was measured by ELISA over a period of 6 days (Fig. 1, top and bottom respectively). There was no apparent difference within this period in either solution or on TCPS between PU and PA. Lower absorbance of RG-I was observed in solution (Fig. 1 top) than on coated TCPS plates (Fig. 1 bottom). These suggest that RG-I are stable on TCPS plates.

### Galectin-3 binding affinities of PU and PA

**Fig. 2:**
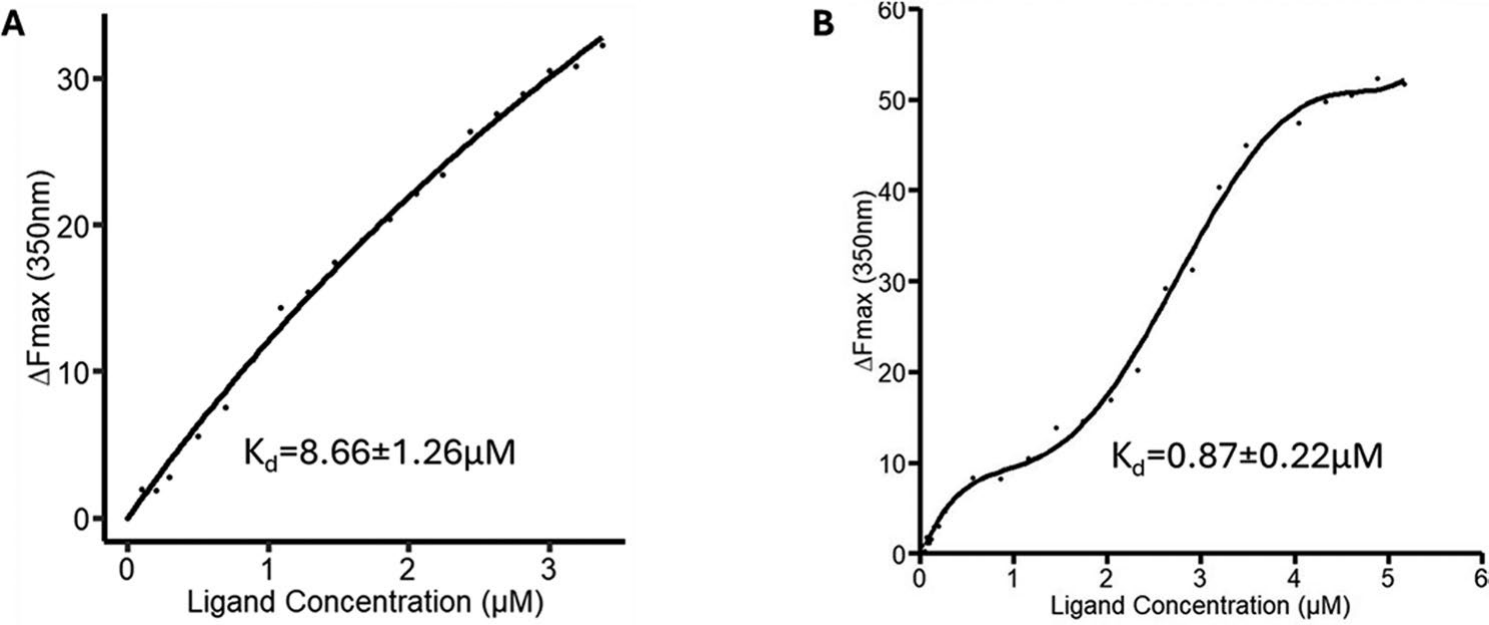
Analysis of PU and PA binding to galectin-3. Binding of PU and PA was measured by TFS. Changes of tryptophan florescence intensity of galectin-3 with increasing concentrations of PU (**A**) or PA (**B**) were recorded, and the binding affinity of PA and PU to galectin-3 was determined using a nonlinear curve fitting

To investigate the interaction of PA and PU with galectin-3, the binding affinity of PA and PU to galectin-3 was determined by TFS which measures interaction of PA and PU with galectin-3 in non-labelled, completely free condition [22]. TFS analysis showed binding of both PU and PA to galectin-3 fitting into one binding site model. PU bound to galectin-3 with binding affinity K_d_, 8.66 ± 1.26µM (Fig. 2). PA was found to bind to galectin-3 10-times stronger (K_d_, 0.87 ± 0.22µM) than PU. These results indicate that enzymatical treatment of RG-I, which shorten the arabinose and exposes the galactose sidechains of RG-I, increased RG-I binding to galectin-3.

### Differentially expressed genes by hMSCs in response to PA coating

Transcriptomic profiling (Illumina, San Diego, CA, USA) identified 42 genes differentially expressed between PA and control (non-coated TCPS) with FDR < 0.1 (10%) (Supplementary data, Table S1). Twenty of these genes were upregulated with FDR < 0.1 and log2FC > 1.5 such as HMGA2 (High Mobility Group AT-Hook 2) and its transcriptional regulator HMGA2-AS1 (HMGA2 Antisense RNA 1), whereas 11 genes were downregulated with FDR > 0.1 and log2FC<-1.5. such as TM9SF1 (transmembrane 9 superfamily member 1). The 42 genes were subjected to in-silico analysis utilising IPA to identify regulatory effects, common pathways and/or gene networks. Galectin-3 was also included in the IPA as it was previously identified to be downregulated in PA-functionalised scaffolds [9].

**Fig. 3:**
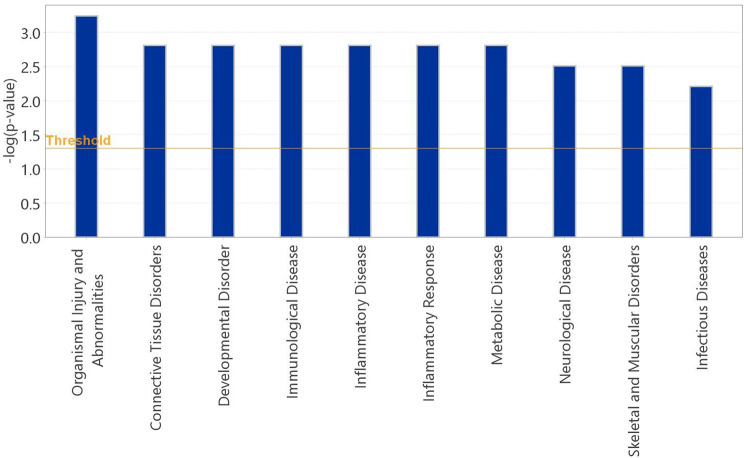
Targeted pathway enrichment analysis. Pathway enrichment analysis for diseases and functions of the differentially expressed genes (DEGs) including galectin-3 showed enrichment for organismal injuries and abnormalities, immunological, inflammatory, metabolic infectious and diseases, skeletal and muscular disorders

Pathway enrichment analysis for diseases and functions of the DEGs including galectin-3 showed enrichment for organismal disorders-abnormalities, connective tissue disorders, immunological and inflammatory diseases, inflammatory response and skeletal muscular disorders (Fig. 3).

**Fig. 4:**
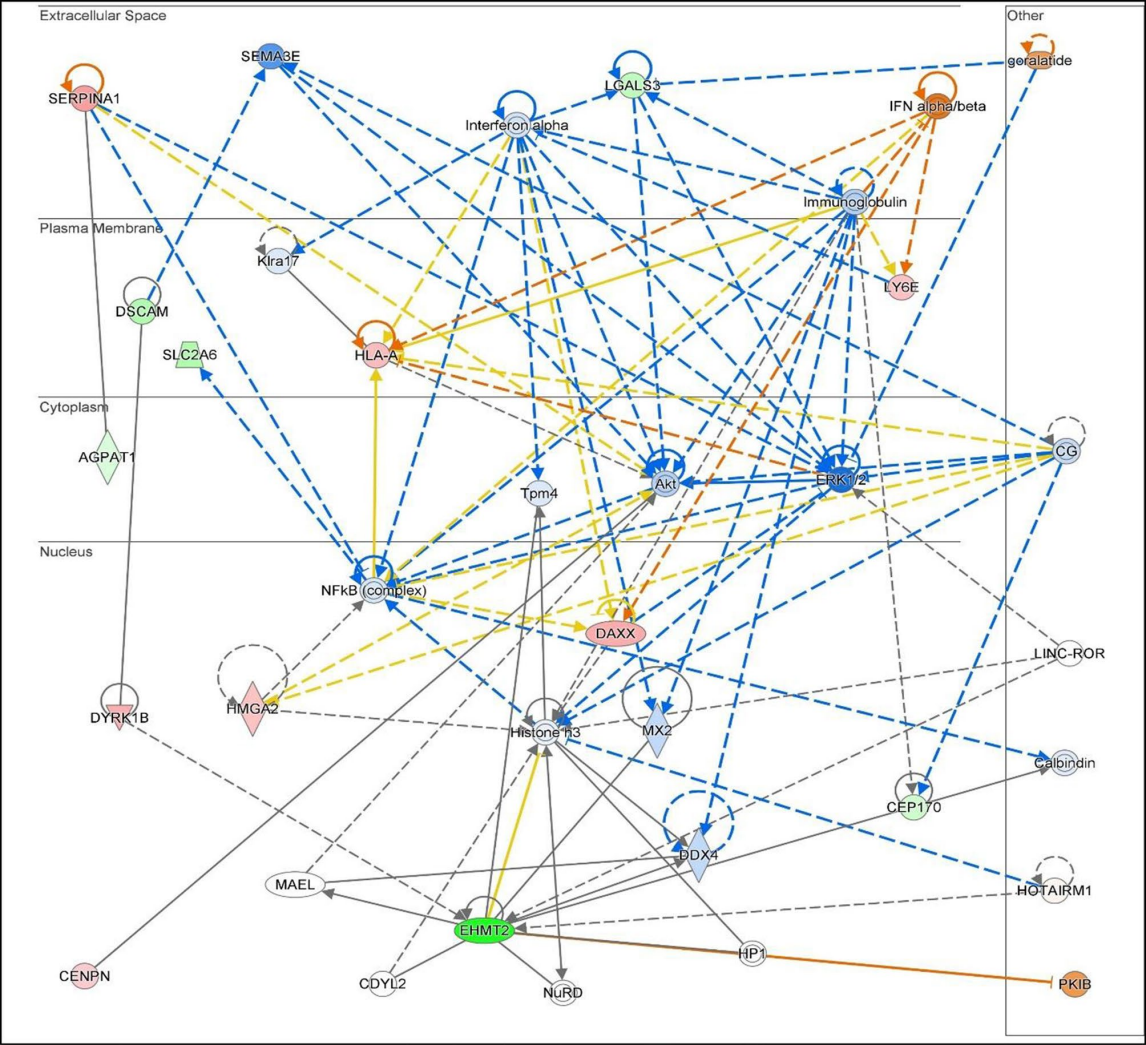
Network 1 gene interactions. Illustration of Network 1 highlighting the gene interactions and prediction of regulations. Predicted activity level is highlighted by Green: downregulated, Red: upregulated. Orange: activated, Blue: inhibited. Predicted relationship between nodes is highlighted by Orange: leads to activation, Blue: leads to inhibition, Yellow: inconsistent findings in relationship to downstream molecule; Grey: effect not predicted

Molecular network analysis linked the DEGs into 3 networks (Table 1 and Supplementary data, Table S2). This network analysis showed the interactions between the DEGs in our dataset together with upstream and/or downstream identified regulatory molecules through IPA. The highest ranked network (ID1, score 59) involved 21 genes identified in this study including galectin-3, linking them to infectious diseases, immune & inflammatory response and cell cycle control. The associated interaction network map in terms of these molecules is shown in Fig. 4. The second network (ID2, score 23) linked 10 of the DEGs with cellular responses to therapeutics, infectious diseases, organismal injury & abnormalities whereas the third network (ID3) had the lowest score, with 4 genes from our study, showing involvement in cancer, cellular assembly organisation, and RNA post-transcriptional modifications. Causal networks between the molecules in the 3 networks are presented in Supplementary data, Table S3. Finally, we identified upstream regulators in relation to the above analysis which includes IFN alpha/beta; DYSF; MYBL1; ADAR; SNORD21, with p-values < 0.05 (Supplementary data, Table S4).

**Table 1:**
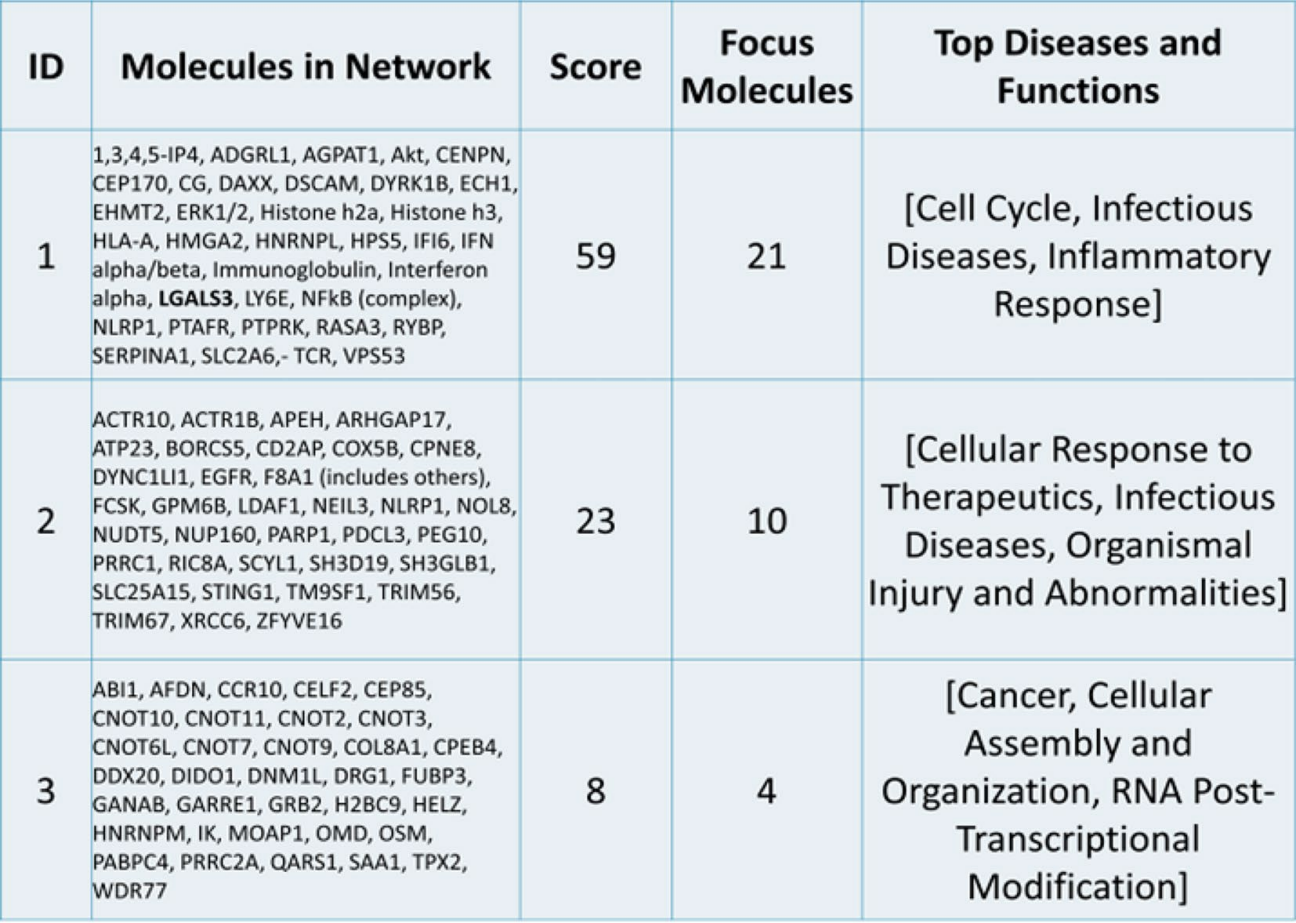
Molecular network analysis. The 42 differential expressed genes and galectin-3 (LGALS3) are linked to 3 molecular networks as identified by IPA

## Discussion

In this study unmodified (PU) showed binding to galectin-3 at low micromolar affinity. Enzymatically removal of the arabinose sidechain from PU, which exposes the galactose-residue on PA RG-I, increased the binding by 10-fold. The in-silico analysis indicates the regulation of modified RG-I (PA) on several genes in relation to inflammatory molecules and cellular pathways, indicating the capability of RG-I on modulation of inflammation.

Galectin-3 is known to play a significant role in modulating the inflammatory response. It can act as both a pro-inflammatory and anti-inflammatory molecule [12, 19, 25–28]. The demonstration of galectin-3 gene interactions in network 1 in this in-silico analysis suggests the interaction of galectin-3 with various inflammatory molecules, linking them to infectious diseases, immune & inflammatory response, and cell cycle control. In this study Gal-3 downregulation by PA may reflect an anti-inflammatory shift favourable for regeneration. In our previous work [9], we showed decreased level of galectin-3 on gene and protein level in osteoblast cell culture on PA-coated scaffolds. Our in-silico analysis, supported our previous findings and identified further inflammatory markers potentially related to galectin-3 such as IFN alpha/beta; DUSF, MYBL1, ADAR, SNORD21, that their relation needs to be confirmed.

One of the most differentially expressed genes in transcriptomic analysis between control and PA coating was HMGA2. HMGA2 encodes a protein that belongs to the high mobility group A (HMGA) family of architectural transcription factors. It plays a role in chromatin organisation and has been implicated in cell proliferation and tumorigenesis [29]. HMGA has also been shown to be involved in bone regeneration and osteoblast regeneration, through expression on MSCs in facial bone ossification sites [30]. However, due to the lack of evidence between these two genes with PA, we can only infer that both genes can play complimentary roles in promoting bone regeneration, but this would require further investigation.

Another gene that has been identified from the in-silico analysis is TM9SF1, involved in the regulation of autophagy, as an autophagosome-inducing gene. Overexpression of TM9SF1 has been shown to induce high levels of autophagosome formation, while TM9SF1 in a knockout mouse model of acute lung injury has been found to decrease inflammation by enhancing autophagy [31]. In addition, a recent study found that TM9SF1 together with its interactor EBAG9 play a physiological role in bone maintenance by promoting autophagy [32]. Autophagy plays a crucial role in bone homeostasis and regeneration [33] therefore we can infer that this gene could potentially influence bone cell function and regeneration processes.

Research for the potential of plant-based scaffolds for bone regeneration has gained significant attention recently, with particular attention on their ability to modulate inflammatory responses and provide a sustainable alternative to synthetic materials. Recent studies have reported 3D-bioprinted pectin-based hydrogels [9, 18], that has shown good printability, mechanical properties, and biocompatibility, making it a promising alternative to synthetic polymers. This is supported by the physical and biological characterisation of the pectin-based hydrogel, which demonstrated its ability to promote chondrocyte proliferation and maturation, maintain cell viability, and support the formation of the extracellular matrix [9]. The use of pectin RG-I, extracted from agricultural side-streams, as a bioink for 3D bioprinting has shown promising properties for potential use in repairing osteochondral defects due to its anti-inflammatory effect and fibrosis inhibition [9]. The interaction of plant-derived enzymatically modified RG-I (PA) with hMSCs suggested in this study a potential mechanism of action, however, the results need to be confirmed and investigated with RG-I functionalised scaffolds for bone regeneration. This constitutes a limitation of this study together with the small number of participants, although the isolated hMSCs were cultured in triplicates. In addition, both our participants were females, limiting the generalisation of our results. Studies have shown that donor sex is an important factor contributing to MSC heterogeneity and potency, emphasizing that it will be beneficial to determine the MSC donor sex depending on the targeting disease in future clinical trials and/or animal studies [34, 35]. Nevertheless, this study suggests the potential of plant-derived polysaccharides as a sustainable alternative to synthetic polymers for bone reconstruction. The great advantage of RG-I is its source, as it is isolated from side streams of potato starch production, that is a byproduct from food industry and might contribute to development of novel strategy for production of sustainable biomaterials for tissue engineering to reduce the environmental impact and improve the sustainability of healthcare.

## Conclusions

Modulating the inflammatory response could be beneficial for bone regeneration, and an anti-inflammatory response is particularly desirable in the later stages of healing. Galectin-3 plays a crucial role in this process by modulating inflammation and influencing the differentiation and function of bone cells via binding to various galactose-terminated cell surface glycans. This study suggests that potato-derived, enzymatically modified RG-I (PA) can potentially modulate the expression of various genes including galectin-3 in hMSCs, indicating that RG-I may have the potential to be further investigated in the modulation of bone regeneration.

## Supplementary Information

Below is the link to the electronic supplementary material.

Supplementary Material 1

## Data Availability

All data generated and analysed during this study are included in this published article and its supplementary information files. The raw data generated from the transcriptomic profiling (Illumina) are available at the European Nucleotide Archive (ENA) Sequence Read Archive (SRA), accession number PRJEB89424 and secondary accession number ERP172451.

## Abbreviations

BH: Benjamini-Hochberg
DE: Differentially expressed
DEGs: Differentially expressed genes
FBS: Foetal bovine serum
FDR: False-discovery rate
HMGA: High mobility group A
HMGA2-AS1: HMGA2 Antisense RNA 1
hMSC: Human mesenchymal stem cells
ID1: Identification 1 (highest ranked network)
ID2: Identification 2 (second ranked network)
ID3: Identification 3 (third ranked network)
IPA: Ingenuity pathway analysis
kDA: Kilodalton
LGALS1: Galectin-1
LGALS3: Galectin-3
MEM: Culture medium
ml: Millilitter
µM: Micromolar
MSC: Mesenchymal stem cells
Mw: Molecular weight
nm: Nanometer
PA: Arabinose-deficient form rhamnogalacturonan-I
PBS: Phosphate-buffer saline
PU: Unmodified rhamnogalacturonan-I
RG-I: Rhamnogalacturonan-I
TCPS: Tissue culture polystyrene surface plates
TFS: Tryptophan fluorescence spectroscopy
TM9SF1: Transmembrane 9 superfamily member 1
